# Supportive care and information needs in relation to quality of life among patients with breast cancer and gynaecological cancer during the time of treatment

**DOI:** 10.1007/s00404-024-07805-7

**Published:** 2024-11-22

**Authors:** Saskia-Laureen Herbert, A. S. Payerl, M. Prange, S. Löb, J. Büchel, A. Scherer-Quenzer, M. Kiesel, A. Wöckel, H. Faller, K. Meng

**Affiliations:** 1 Department of Obstetrics and Gynaecology, University Medical Centre Würzburg, Josef-Schneider-Straße 8, 97080 Würzburg, Germany; 2 Department of Obstetrics and Gynaecology, Regiomed Kliniken, Coburg, Germany; 3 Department of Clinical Epidemiology and Biometry, University Würzburg, Würzburg, Germany

**Keywords:** Breast cancer, Gynaecological cancer- needs, Quality of life, Health care providers

## Abstract

**Purpose:**

Although therapy and psychosocial care for patients with breast cancer and gynaecological cancer has improved in the last years, there are still many issues that require further investigation. Unmet supportive care needs can lead to a lower adherence to treatment and a lower quality of life. Patients’ needs seem to be highest during the time of treatment. Thus, this study investigated needs and quality of life.

**Methods:**

In this German prospective study, we enrolled 292 patients with breast cancer and gynaecological cancer during the time of treatment. Data on needs were assessed using instruments that had proven feasible in earlier studies. Data on quality of life (QoL) were assessed using the European Organization for Research and Treatment of Cancer QoL Core Questionnaire (EORTC QLQ-C30). We investigated correlations between needs and sociodemographic data as well as quality of life.

**Results:**

Among all cancer entities we observed that 150 patients (51.5%) showed unmet information needs, 221 patients (75.7%) showed at least one high supportive care need, and 91 patients (31.2%) had psychological care needs. Data showed statistically significant correlations between these needs and sociodemographic data as well as quality of life. These correlations generally showed small to medium effect sizes. Older women showed less supportive care needs (r = − 0.24; p < 0.001), (r = − 0.15; p = 0.010). Furthermore, recruitment after surgery was associated with statistically significant higher information needs (r = 0.14; p = 0.015), whereas recruitment during chemotherapy was associated with statistically significant less information needs (r = − 0.15; p = 0.013). Positive correlations were shown for the level of received information and physical functioning (r = 0.12; p = 0.047), social functioning (r = 0.16; p = 0.009) and global quality of life (r = 0.19, p = 0.002) as well as satisfaction with information and physical (r = 0.16; p = 0.006), social (r = 0.24; p < 0.001), cognitive functioning (r = 0.14; p = 0.017) as well as global quality of life (r = 0.25; p < 0.001). Negative correlations were reported for information needs and emotional functioning (r = − 0.12; p = 0.035) and global quality of life (r = − 0.15; p = 0.011). Supportive care needs also correlated negatively with physical (r = − 0.23; p < 0.001), role (r = − 0.23; p < 0.001), emotional (r = − 0.35; p < 0.001), cognitive (r = − 0.24; p < 0.001), social functioning (r = − 0.30; p < 0.001), and global quality of life (r = − 0.35; p < 0.001). Also, patients with at least one high supportive care need correlated negatively with role (r = − 0.15; p = 0.014), emotional (r = − 0.23; p < 0.001), social functioning (r = − 0.30; p = 0.001), and global quality of life (r = − 0.35; p < 0.001). There was no statistical significance concerning cancer side. Thus, both groups are reported together. Furthermore, there was no statistical significance concerning disease status.

**Conclusion:**

Overall, this study highlights the importance of tailored information and supportive care interventions. Addressing these needs, particularly in terms of information provision and psychosocial support, could lead to improved quality of life and better overall patient outcomes.

## What does this study add to the clinical work

**Table:** 

Providing tailored information and supportive care interventions is essential for improving quality of life in patients with breast cancer and gynecological cancer. Meeting these needs, particularly during active treatment phases, may positively impact overall well-being.	

## Introduction

The most common cancer among women is breast cancer. About 30% of female patients with cancer suffer from breast cancer in Germany. The fifth leading localisation for cancer is the uterus, especially corpus uteri. More than 3% of female cancer is caused by ovarian cancer. Summing up about 40% of female patients with cancer are affected by gynaecological malignancies and breast cancer. Since patients with cancer are confronted with physical symptoms, psychological and psychosocial problems especially during treatment, health care providers have to focus on patients’ needs. Unmet supportive care needs can lead to reduced quality of life [1] and decreased compliance, with worsened outcomes [2]. Hence, in this study we investigated supportive care needs and quality of life among women with breast cancer and gynaecological cancer during the period of treatment.

Supportive care can be defined as the provision of necessary support meeting informational, physical, social, and emotional needs. These needs can be felt differently and differ between patients [3]. “During the time of treatment” is defined as a visit in hospital for either surgery or neoadjuvant/adjuvant/palliative systemic therapy, which allows distinguishing local and systemic issues. Supportive care needs can be identified by health care providers. Felt needs and professionally captured needs are not always matching [4].

Treatment of breast cancer and gynaecological cancer shows acute as well as long-term side effects. Severity is dependent on personal factors such as age and comorbidities as well as the type of therapy [5]. Patients with breast cancer and gynaecological cancer can be affected by general symptoms as well as specific toxicities such as pain, nausea, vomiting, fatigue, insomnia, alopecia, stomatitis, myelosuppression, lymphedema, premature menopause and thromboembolism caused by therapy [6–8]. These sequelae require supportive care. Studies have already shown that patients with cancer have unmet supportive care needs [9, 10]. Besides, psychological and social complications can also affect patients with cancer and lead to decreased quality of life. Many patients with breast cancer suffer from distress caused by therapy, fear of recurrence, different body image as well as changes in sexuality [11]. According to that, studies have shown need for support dealing with emotional and existential problems both for breast [12] and gynaecological cancer [13]. Although effective interventions improved quality of life in patients with breast cancer during the last years there are still many issues that require further consideration in order to improve quality of life [14]. For patients with gynaecological cancer, it is already known that treatment with chemotherapy still leads to a lower quality of life and lower involvement in social activities [15].

Need for additional medical information has also been reported for both breast [16, 17] and gynaecological cancer [18, 19]. Machacek et al. demonstrated the importance of information provision. They showed lower levels of worry and pain using brochures and communication training for the interaction with patients undergoing breast biopsy [20]. Furthermore, the need for support concerning communication [12] and getting in touch with other cancer patients has been reported.

Improving supportive care during treatment of breast cancer and gynaecological cancer is of great importance concerning management of treatment related symptoms, increasing adherence to treatment, and addressing psychological and social aspects of cancer and cancer treatment. Hence, meeting supportive care needs should be a part of interdisciplinary cancer care requiring education of health care providers and patient awareness of supportive care services.

In German studies on breast cancer, baseline levels of information received predicted subsequent quality of life and depressive symptoms. However, similar studies are lacking in gynecological cancer.

Thus, this study had three aims: (1) to identify information and supportive care needs and to explore to which extent they are met in patients with breast cancer compared to patients with gynaecological cancer during the time of treatment; (2) to examine associations between information/supportive care needs and sociodemographic and medical parameters; (3) to explore the associations between information/supportive care needs and quality of life.

## Methods

This study was approved by the Ethics Committee of the Medical Faculty of the University of Würzburg (file number 282/16). The study complied with the Declaration of Helsinki. All participants provided written informed consent.

### Study participants and setting

Time of recruitment was during treatment. Patients came to hospital for surgery, chemotherapy or because of other reasons such as complications of treatment.

Cancer patients were enrolled at two hospitals. Recruitment process was independent of type of treatment and prognosis. Inclusion criteria comprised histologically proven breast cancer or gynaecological cancer (cervix uteri, corpus uteri, ovary), female gender, age of 18 years or older and sufficient German language skills. Exclusion criteria comprised the comorbidity of serious mental disorders and serious impairment of eyesight or auditory sense (without adjustment). 555 patients eligible were asked to participate. 306 gave informed consent and completed the questionnaire, but 14 patients had to be excluded (e.g., high rate of missing data). Therefore, the final sample comprised 292 patients.

### Measures

#### Information needs

To assess information needs, we used instruments that had proven feasible in earlier studies [4, 21, 22]. Patients report their level of information and unmet information needs using eight items representing different topics: cancer diagnosis/disease, chance of cure/recovery, likely course of the disease, possible treatment options, risks/side effects of treatments, dealing with disease-related stress, psychological support offers, and aftercare. Patients were asked to evaluate to which degree they felt informed about the respective topic, using 5-point Likert scales ranging from “not at all informed (1)” to “very much informed (5)” (score “level of information received”: Cronbach`s alpha = 0.87). In addition, for each item, patients were asked to indicate whether they wish additional information about the respective topic, using a dichotomous response format (“yes”, “no”). Items are summed up to an information needs score, respectively.

Overall satisfaction with information was assessed using two items (“How satisfied are you overall with your level of information?”, “How satisfied are you with the amount of information received?”). Items were answered on a 7-point numerical rating scale, with higher scores indicating higher satisfaction. Reliability was high (Cronbach's alpha = 0.95).

#### Supportive care needs

We used an instrument with 12 items developed in a prior study [23]. Each item comprises supportive care needs on a 4-point Likert scale (1 = no need, 2 = low need, 3 = medium need, 4 = strong need): alleviation of physical symptoms, enhancement of health behaviours, professional psychosocial support, counselling regarding partnership and sexuality, opportunities to talk to other people, contacting other cancer survivors, medical information, access to a coordinator of treatment and aftercare, legal and financial counselling, managing household and everyday activities, return to work, and transportation to aftercare examinations. In addition to assessing the strengths of needs in individual domains, items were summed up into an overall score (“supportive care needs”; Cronbach`s alpha = 0.87).

The variable “high supportive care needs” indicates the number of patients with at least one high supportive care need.

#### Psychosocial care needs

We used a single item to determine the need for psychosocial support, with dichotomous response format (“yes”, “no”): “Do you have a need for psychosocial support?” [4, 24].

#### Quality of life (Qol)

Patients completed the German version of the European Organization for Research and Treatment of Cancer Quality of Life Questionnaire Core 30 (EORTC QLQ-C30) [25]. The QLQ-C30 contains five functional scales (physical, social, role, cognitive, and emotional), three symptom scales (fatigue, pain, nausea and vomiting), a global health status, and six single items (dyspnea, loss of appetite, insomnia, constipation, diarrhea and financial difficulties). All the scales are transformed into a range from 0 to 100, with higher scores reflecting better health status (functional scales, global health status) or a high level of symptomatology (symptom scales). Cronbach`s alpha in our data was 0.69 to 0.93.

Sociodemographic characteristics were assessed by self-report and clinical data were obtained from the patient charts.

### Statistical analysis

For the sample description and presenting the needs we used descriptive analyses (frequencies, percentages, means, and standard deviations). Chi square test and Mann–Whitney-U-test were used to check for statistical significance. Spearman correlations for sociodemographic and medical parameters as well as quality of life were performed. Correlations coefficients of 0.1/0.3/0.5 were regarded as small/medium/large.

A two-sided p < 0.05 was considered significant. Missing values were excluded by pairwise deletion. All analysis was performed using IBM SPSS 26.0 (Armonk, NY, USA) software.

## Results

### Sample

Table 1 shows the sociodemographic and clinical sample characteristics of the 292 participants. Seventy-five percent were diagnosed with breast cancer and 25% with a gynaecological cancer. The mean age of the participants was 58.2 years (SD = 12.7; range 26 to 91). About 69% were living with a partner. 64% received more than basic education. 51% of the participants were recruited during a visit for surgery and 39% during a visit for chemotherapy. About two thirds of the patients were diagnosed at stage I or II. Nearly half of the patients already showed complete remission at the time of recruitment. More than 90% had to undergo surgery, and nearly 70% chemotherapy or radiotherapy, respectively.

**Table 1:** Characteristics

	Total N = 292	Breast Cancer n = 219	Gynecological Cancer n = 73	P-value
Age (years; n = 290), mean (SD)	58.2 (12.7)	58.7 (13.0)	57.0 (11.7)	0.32
Marital status (n = 291), n (%)				0.23
Unmarried	35 (12.0)	22 (10.0)	13 (17.8)	
Married	184 (63.0)	141 (64.6)	43 (58.9)	
Divorced	27 (9.2)	21 (9.6)	6 (8.2)	
Widowed	44 (15.1)	33 (15.1)	11 (15.1)	
Living with partner (n = 280), n (%)	201 (68.8)	155 (70.1)	46 (67.1)	0.51
Education (n = 281), n (%)				0.31
Less than junior (< 10y)	91 (31.2)	70 (32.0)	21 (28.8)	
Junior (10y)	114 (39.0)	83 (37.9)	31 (42.5)	
Senior (high-scholl graduate)	74 (25.3)	55 (25.1)	19 (26.0)	
No graduation/ other	2 (0.6)	2 (0.9)	0 (0.0)	
Cancer site (n = 292), n (%)				
Breast	219 (75.0)	219 (100.0)	-	
Ovary	31 (10.6)	–	31 (42.5)	
Endometrium	15 (5.1)	–	15 (20.5)	
Cervix	27 (9.2)	–	27 (37.0)	
Time of recruitment (n = 289), n (%)				0.01
Surgery	148 (50.7)	119 (54.3)	29 (39.7)	
Chemotherapy	115 (39.4)	76 (34.7)	39 (53.4)	
Other	26 (8.9)	22 (10.0)	4 (5.5)	
Missings	3 (1.0)	2 (0.9)	1 (1.4)	
Disease status (n = 265), n (%)				< 0.001
Remission	136 (46.6)	110 (50.2)	26 (35.6)	
Partial remission	31 (10.6)	11 (5.0)	20 (27.4)	
Progressive	38 (13.0)	25 (11.4)	13 (17.8)	
Stable	36 (12.3)	27 (12.3)	9 (12.3)	
Inconclusive evaluation	24 (8.2)	21 (9.6)	3 (4.1)	
Missings	27 (9.2)	25´(11.4)	2 (2.7)	
Disease stage (n = 254), n (%)				< 0.001
Stage I	97 (33.2)	75 (34.2)	22 (30.1)	
Stage II	95 (32.5)	84 (38.4)	11 (15.1)	
Stage III	45 (15.4)	20 (9.1)	25 (34.2)	
Stage IV	17 (5.8)	12 (5.5)	5 (6.8)	
Missings	38 (13.0)	28 (12.8)	10 (13.7)	
Past/ current/ future treatment (n = 292), n (%)				
Surgery	269 (92.1)	205 (93.6)	64 (87.7)	0.62
Radiotherapy	202 (69.2)	176 (80.4)	26 (35.6)	< 0.001
Chemotherapy	204 (69.9)	146 (66.7)	58 (79.5)	0.04

Absolute numbers, means and p-values are presented for the total sample and both groups of cancer separately. Statistical significance was assumed for p-values < 0.05. P-values are from chi^2^-tests. These tests were not performed for each category. Treatment describes the percentage of patients undergoing specific therapy before/during/after participating this study

Women with breast or gynaecological cancer differed regarding time of recruitment (p = 0.014), disease status (p < 0.001), disease stage (p < 0.001), and treatment, i.e., undergoing radiotherapy (p < 0.001).

### Information needs

89.9% of patients with breast cancer (bc) and 76.8% of patients with gynaecological cancer (gc) felt quite or very much informed about diagnosis/disease, 70.8% (bc)/ 54.8% (gc) about chances of recovery, 67.2% (bc)/ 54.8% (gc) about course of disease, 66.6%(bc)/ 50.7% (gc) about different treatment options, 67.2% (bc)/ 69.9% (gc) about risks/ side effects, 59.8% (bc)/ 56.2% (gc) about coping with disease, 77.1% (bc)/ 60.3% (gc) about psychological support and 50.2% (bc)/ 45.2% (gc) about aftercare.

**Fig. 1:**
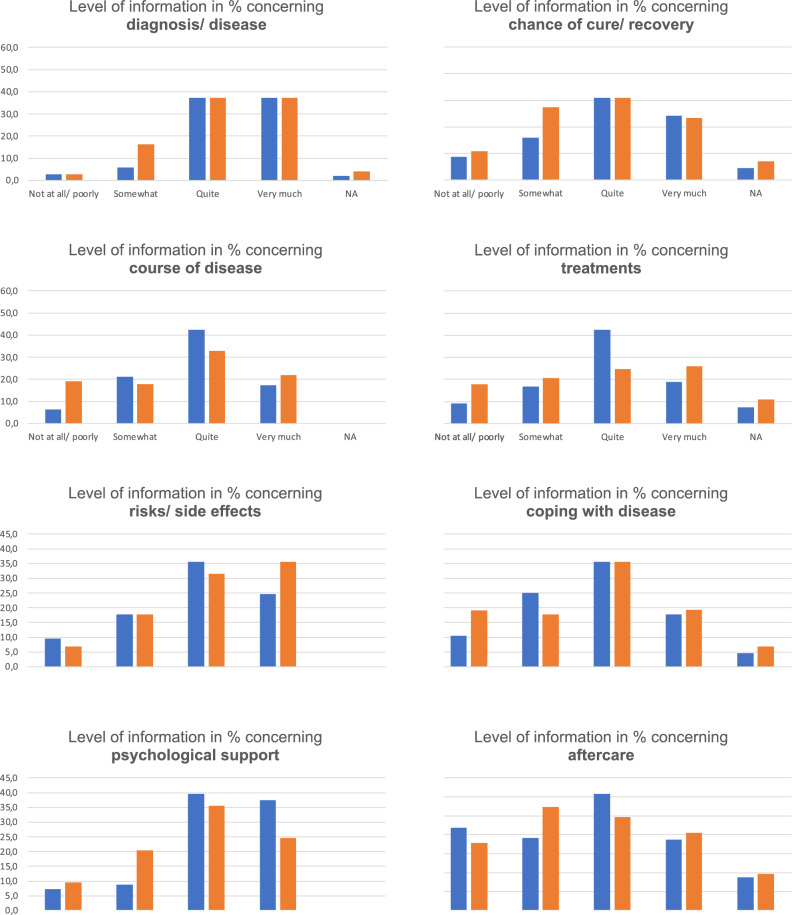
Level of information. Patients’ self-reported level of information received about various aspects of their disease by cancer type. Numbers represent percentages

Patients with breast cancer showed higher levels of information received concerning diagnosis/disease (p = 0.029), course of disease (p = 0.008), treatment (p = 0.013) and psychological support (p = 0.041) compared to patients with gynaecological cancer (Fig. 1).

**Fig. 2:**
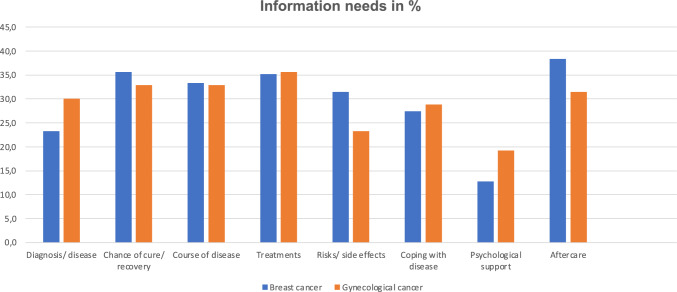
Information needs. Patients’ self-reported need for information about various aspects of their disease by cancer type. Numbers represent percentages. Missings were excluded

Patients were asked to indicate whether they wish additional information. About one third of the participants showed information needs concerning diagnosis/disease, chance of recovery, course of disease, treatment options, adverse effects and coping with disease and aftercare (Fig. 2). There was no significant difference between the groups.

All in all, both patients with breast and gynaecological cancer showed similar scales of level of information received, satisfaction with information, supportive care needs and information needs (Table 2).

**Table 2:** Scales

	Breast cancer		Gynaecological cancer		
	Mean	SD	Mean	SD	p-value
Scale information needs	2.4	2.8	2.5	3	0.837
Scale level of information received	3.8	0.7	3.7	0.8	0.295
Scale satisfaction with information	5.4	1.3	5.2	1.4	0.329
Scale supportive care needs	2.4	0.7	2.5	0.8	0.483

Statistical significance was assumed for p-values < 0.05

Patients’ scales for supportive care need, information need, satisfaction with information and level of information received. Mean values are presented (0–12). Scales are shown separately for each cancer type

### Supportive care needs

**Fig. 3:**
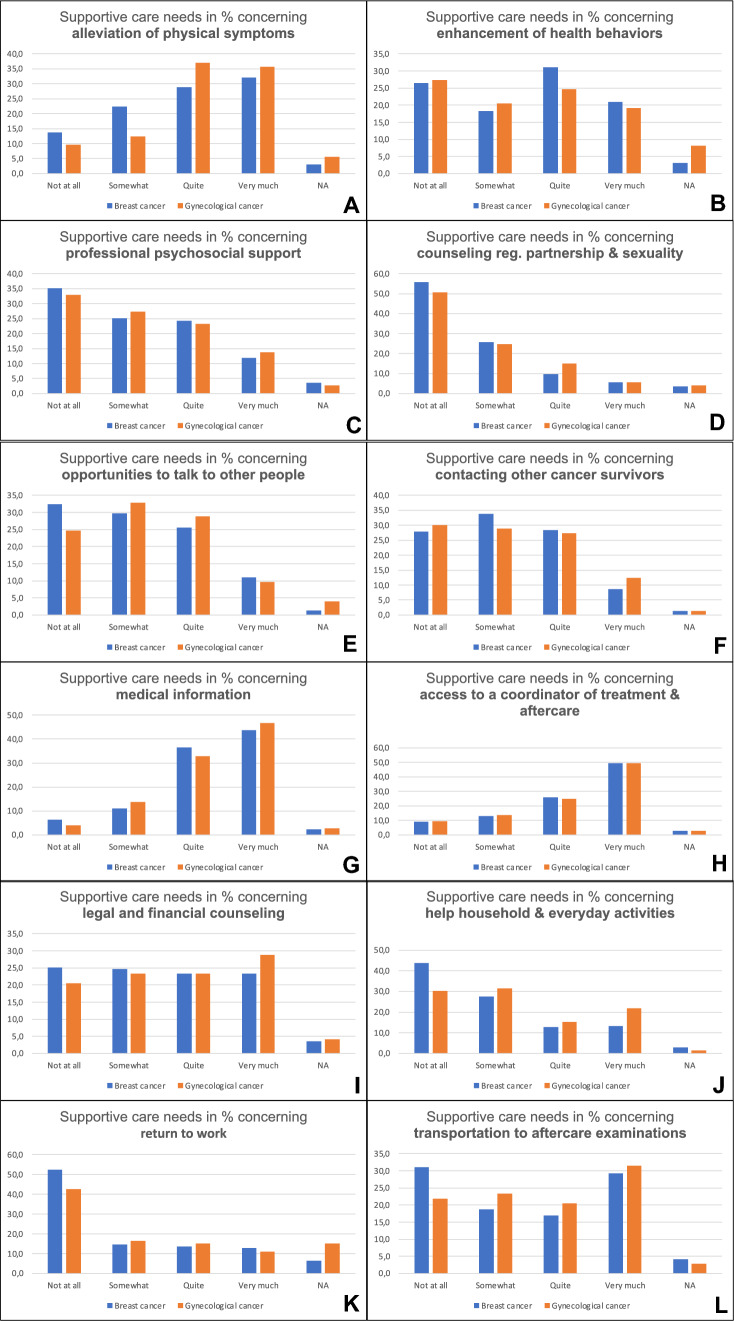
Supportive care needs. Patients’ self-reported level of supportive care needs. Aspects are shown separately for each cancer type. Numbers represent percentages

Patients with breast and gynaecological cancer reported quite or very much supportive care needs concerning transportation to aftercare examinations, legal and financial counselling and enhancement of health behaviours in nearly 50%, concerning access to a coordinator of treatment and aftercare in more than 70%, concerning medical information in about 80%, concerning opportunities to talk to other people and alleviation of physical symptoms in about 60% (Fig. 3).

Supportive care needs did not differ between both cancer groups (Fig. 3).

### Psychosocial care needs

**Fig. 4:**
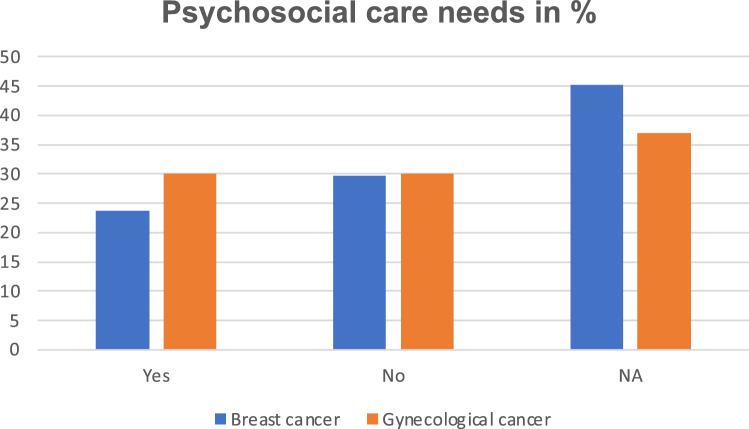
Psychological care need. Patients’ self-reported need of psychological care. Numbers are presented in percentage. Scales are shown separately for each cancer type

23.7% of patients with breast cancer and 30.1% of those with gynaecological cancer reported a need for psychosocial care. There was no difference between both groups (p = 0.42). However, 126 women did not answer this question (Fig. 4).

### Associations between needs and sociodemographic as well as medical parameters

Results are summarized in Table 3. Since there was no statistical significance concerning cancer side both groups are reported together. Furthermore, there was no statistical significance concerning disease status (curative vs. metastasis).

**Table 3:** Spearman correlations

	Need scales	Information needs	Level of received info	Satisfaction info	Supportive care needs	High supportive care needs
**A**						
Inter-correlation of variables of each domain	Information needs	–	− 0.320**	− 0.338**	0.158**	0.201**
	Level of received info	− 0.320**	–	0.747**	− 0.118	− 0.119*
	Satisfaction info	− 0.338**	0.747**	–	− 0.148*	− 0.157**
	Supportive care needs	0.158**	− 0.118	− 0.148*	–	0.591**
	High supportive care needs	0.201**	− 0.119*	− 0.157**	0.591**	–
**B**						
Age	–	− 0.036	− 0.017	0.072	− 0.240**	− 0.150*
Living with partner	(yes; no)	− 0.044	0.027	− 0.015	0.064	0.095
Education	(no graduation/ less than junior/ junior/ senior)	0.067	− 0.106	− 0.062	0.024	0.040
Time of recruitment	Surgery	0.142*	0.030	0.075	− 0.009	0.056
	Chemotherapy	− 0.145*	− 0.026	− 0.107	0.033	0.005
Disease stage	(I–IV)	− 0.015	− 0.013	0.053	− 0.024	− 0.001
Disease status	Remission	− 0.015	0.053	0.030	− 0.095	0.039
	Partial remission	− 0.062	0.048	− 0.014	0.098	0.045
	Stable	− 0.014	0.012	0.018	− 0.063	− 0.106
	Progressive	− 0.069	− 0.084	− 0.100	− 0.032	− 0.091
Metastasis	(yes; no)	0.008	0.089	0.027	− 0.037	0.059
Cancer side	(Breast cancer; gyn. Cancer)	− 0.012	− 0.032	− 0.088	0.066	0.029
**C**						
EORTC QLQ− C30	Physical functioning	− 0.068	0.120*	0.161**	− 0.233**	− 0.081
	Role functioning	− 0.105	0.074	0.091	− 0.226**	− 0.146*
	Emotional functioning	− 0.124*	0.089	0.115	− 0.352**	− 0.225**
	Cognitive functioning	− 0.093	0.117	0.141*	− 0.241**	− 0.091
	Social functioning	− 0.113	0.158*	0.241**	− 0.302**	− 0.187**
	Fatigue	0.113	− 0.143*	− 0.199**	0.270**	0.158**
	Nausea	0.058	− 0.075	− 0.128*	0.203**	0.086
	Pain	0.113	− 0.042	− 0.060	0.268**	0.091
	Global QoL	− 0.148*	0.190**	0.246**	− 0.346**	− 0.195**

Shown is the Spearman correlation between supportive care/ information needs and A: intercorrelations, B: sociodemographic parameters/ medical information as well as C: health-related outcomes (quality of life). The questionnaire variables include unmet need info (suffering from at least one unmet need concerning information), informedness (the scale for level of information received), satisfaction info (satisfaction with received information), scale supportive care needs (the scale for level of supportive care needs) and high supportive care needs (at least one high supportive care need was reported). Health related outcomes comprise physical/role/emotional/ cognitive and social functioning as well as fatigue, nausea, pain and Global QoL. Note: Correlation coefficients of 0.1/0.3/0.5 were regarded as small/medium/large

*p < 0.05

**p < 0.01

***p < 0.001

Information needs, satisfaction scores and supportive care needs scores showed no consistent and only significant, but small correlations with age, and time of recruitment. However, older women showed less supportive care needs (r = − 0.24; r = − 0.15). Furthermore, recruitment after surgery was associated with more information needs (r = 0.14), whereas recruitment during chemotherapy was associated with less information needs (r = − 0.15).

### Association between needs and quality of life

Results are summarized in Table 3. Since there was no statistical significance concerning cancer side both groups are reported together. Furthermore, there was no statistical significance concerning disease status (curative vs. metastasis).

Information needs, satisfaction scores and supportive care needs scores showed small to medium significant correlations with physical, role, emotional, cognitive, and social functioning as well as fatigue, nausea, pain and global QoL.

Positive correlations were shown for the level of received information and physical (r = 0.12), social functioning (r = 0.16) and global quality of life (r = 0.19) as well as satisfaction of information and physical (r = 0.16), social (r = 0.24), cognitive functioning (r = 0.14) as well as global quality of life (r = 0.25). Furthermore, we observed positive correlations between supportive care needs and fatigue (r = 0.27), nausea (r = 0.20) as well as between patients with at least one high supportive care need and fatigue (r = 0.16).

Negative correlations were reported for information needs and emotional functioning (r = − 0.12), global quality of life (r = − 0.15) as well as between level of received information and fatigue (r = − 0.14). Supportive care needs also correlated negatively with physical (r = − 0.23), role (r = − 0.23), emotional (r = − 0.35), cognitive (r = − 0.24), social functioning (r = − 0.30), and global quality of life (r = − 0.35). Also, patients with at least one high supportive care need correlated negatively with role (r = − 0.15), emotional (r = − 0.23), social functioning (r = − 0.30), and global quality of life (r = − 0.35). Furthermore, we showed a negative correlation between satisfaction with information and fatigue (r = − 0.210), nausea (r = − 0.13). (Table 3).

## Discussion

In this study, we analysed needs and quality of life of patients with breast cancer and gynaecological cancer during the time of treatment. Overall, the results suggest that cancer patients still experience an unacceptable level of unmet needs which can reduce quality of life.

The level of information is quite good. But the level of information needs is still remarkable. We observed that for some aspects more patients with gynaecological cancer felt poorly/not at all informed about different aspects of their disease. However, in both groups about one third of patients showed various information needs concerning diagnosis/disease, chance of cure/recovery, course of the disease, treatment, risks/side effects, coping with the disease, and aftercare. Obviously, a mismatch in information provision and information need can be observed in both groups. Patients with breast cancer and gynaecological cancer expressed similar information needs which is in line with findings of other studies. [16]. It is already known that information is of great importance for patients with breast cancer and gynaecological cancer [16, 26]. Information-seeking can be observed among cancer patients as a strategy for coping and reducing stress [27]. Unmet information needs can result in more symptoms of anxiety and depression [28]. Hence, there is a strong need for improvement of information provision.

In our study, more gynaecological cancer patients felt poorly/not at all informed about coping with the disease, treatment, and course of the disease. These findings may reflect the worse prognosis of some gynaecological cancers, which may constitute a burden to provide these patients with adequate information [29]. Additionally, different information needs can be observed throughout the patients´ cancer journey [30]. Therefore, adjustment of information provision to the individual needs of cancer patients is required.

Nevertheless, interpretating data at the time of recruitment (during therapy) needs to be considered as well. Literature shows that unmet information needs are higher during treatment and decrease post-treatment in general [26]. It can be discussed if this phenomenon reflects the normal way of information status through the cancer journey. However, since information needs can still be found among breast cancer survivors five years after diagnosis [31], information provision before and during treatment needs to be reconsidered. Interestingly, we observed that patients undergoing surgery showed more information needs, whereas patients undergoing chemotherapy had less information needs at the time of recruitment. Usually, patients on chemotherapy show high needs unchanged after a few cycles [32].

Concerning supportive care, 60% up to 70% of patients with breast cancer and gynaecological cancer showed needs quite and very much concerning alleviation of symptoms, medical information, access to a coordinator of treatment. Only for patients with gynaecological cancer we observed supportive care needs concerning legal and financial counselling as well as help with household. It is already known that cancer patients experience high levels of unmet needs [33]. According to this study, literature shows the impairment of work, home management, sleep, physical and social activities caused by diagnosis and treatment in cancer patients [34, 35]. Therefore, supportive care needs seem to be highest and most diverse during time of treatment [36]. In this study, supportive care needs are higher than information needs. Other older studies found the highest level of unmet needs in the information domain [37]. This may reflect an improvement of provision and quality of information given to patients with cancer.

Taken together, identification and management of supportive care needs is a crucial aspect of health care for patients with cancer.

These needs and quality of life showed small to medium correlations. More information needs correlated with lower emotional functioning and lower global quality of life. Whereas a higher level of information received correlated with higher quality of life and less fatigue. Appropriate information provision can improve the sense of control and health-related quality of life (HRQoL) [38]. A lack of information can affect mental and physical well-being. In addition, more supportive care needs correlate with lower physical, role, emotional, cognitive and social functioning and global quality of life as well as more fatigue and nausea. This association between unmet needs and reduced QoL is in line with other studies [39–41]. Five years after diagnosis breast cancer patients still suffer from reduced quality of life [42]. This indicates the importance of meeting patients´ needs.

A psychological care need was observed among 24% of patients with breast cancer and 30% of patients with gynaecological cancer. A systemic review from 2023 showed for patients with breast cancer that psychological supportive care needs were mentioned most frequently [43]. In particular, Graf et al. observed a higher need for psychological care if patients showed distress [44]. Thus, psychological well-being should be assessed during consultations [45] as it is already recommended by some guidelines concerning care.

The major aim of this study was the survey and assessment of needs. Using the results of this study could be helpful for healthcare providers. A better understanding of how the disease affects a patient´s life can help clinicians adjusting medical care. The assessment of patients´ needs enables health care providers to identify and meet the needs of cancer patients.

Some limitations need to be discussed. Data represents the views of patients with cancer in two German hospitals which may limit the generalizability of the results. Another limitation is that patients underwent different therapies. The extent of surgery and need for chemotherapy depends on cancer type. Hence, there may be different results in these subgroups, but the respective numbers of patients were too small to investigate these subgroups. However, there are studies which analysed different treatments concerning quality of life. For example, Gilbar compared the quality of life between patients that underwent chemotherapy and patients that rejected chemotherapy. There was no difference observed [46]. Another limitation that must be mentioned is missing data. If data is not collected in a controlled or experimental environment, data collection is regularly accompanied by the problem of missing values. These data gaps harbor the risk of distortions in the analysis which could lead to wrong conclusions and decisions. Statistical software ignores this problem and implicitly assume that the user has supplied a complete data table. Missing data therefore represents one of the fundamental problems of empirical work and is also a problem that cannot be solved by the use of statistical software alone. In this case we left out the missings for calculation which means we have less data. Thus, we had only half data answering the question concerning psychosocial care needs. So, this question could not be answered representatively. Finally, we have to address the limitation of the heterogeneity of our study groups. Our aim was to provide a comprehensive insight into the needs and quality of life of patients with gynecological tumors compared to patients with breast cancer, which admittedly led to a complex data collection process. About 30% of female cancer patients suffer from breast cancer in Germany. About 10% of female cancer patients suffer from gynaecological cancer (corpus uteri, cervix uteri, ovarian cancer). Our two cohorts reflect precisely this distribution (219 patients with breast cancer and 73 patients with gynaecological cancer). The number of patients with different gynaecological entities are unfortunately too small to use subgroups comparing them. Hence, we decided to take these three entities together. Since there was no statistical significance concerning cancer side (breast cancer vs. gynaecological cancer) both groups are reported together. But it has to be kept in mind that there could be differences concerning needs among the different entities analysing bigger cohorts.

## Conclusion

Our data indicate that unmet needs, particularly regarding information and supportive care, are associated with poorer quality of life. By focusing on patient-oriented conversations, healthcare providers can more effectively identify individual concerns and tailor support accordingly. This approach fosters a deeper understanding of each patient’s unique needs, allowing for more personalized care and ultimately improving patient outcomes. Based on the findings of our study, we assume that a more patient-oriented communication approach as part of routine care could be beneficial.

## Data Availability

The datasets generated during and/or analysed during the current study are available from the corresponding author on reasonable request.
